# Promising Therapeutic Impact of a Selective Estrogen Receptor Downregulator, Fulvestrant, as Demonstrated In Vitro upon Low-Grade Serous Ovarian Carcinoma Cell Lines

**DOI:** 10.3390/curroncol29060321

**Published:** 2022-06-01

**Authors:** Kamrunnahar Shanta, Kentaro Nakayama, Mohammad Mahmud Hossain, Sultana Razia, Tomoka Ishibashi, Masako Ishikawa, Hitomi Yamashita, Kosuke Kanno, Seiya Sato, Satoru Nakayama, Yoshiro Otsuki, Satoru Kyo

**Affiliations:** 1 Department of Obstetrics and Gynecology, Faculty of Medicine, Shimane University, Izumo 693-8501, Japan; kamrunnahar.vet@gmail.com (K.S.); likhon.vet@gmail.com (M.M.H.); raeedahmed@yahoo.com (S.R.); tomoka@med.shimane-u.ac.jp (T.I.); m-ishi@med.shimane-u.ac.jp (M.I.); memedasudasu1103@gmail.com (H.Y.); kanno39@med.shimane-u.ac.jp (K.K.); sato_seiya9534@yahoo.co.jp (S.S.); satoruky@med.shimane-u.ac.jp (S.K.); 2 Department of Obstetrics and Gynecology, Seirei Hamamatsu Hospital, Hamamatsu 430-8558, Japan; satoru@sis.seirei.or.jp; 3 Department of Organ Pathology, Seirei Hamamatsu Hospital, Hamamatsu 430-8558, Japan; otsuki@sis.seirei.or.jp

**Keywords:** low-grade serous ovarian carcinoma, estrogen receptor, *PIK3CA*, fulvestrant

## Abstract

Few studies have reported hormonal agent use in the treatment of low-grade serous ovarian carcinomas (LGSOCs), which are chemoresistant. Considering the need for novel effective therapies, we investigated the hormone receptor expression and hormonal inhibition efficacy in LGSOCs. Using immunohistochemistry, we assessed the estrogen receptor (ER) expression status in 33 cases of histologically confirmed serous ovarian tumors, including 10, 11, and 12 cases of LGSOCs, serous borderline tumors (SBTs), and serous cystadenomas (SCAs), respectively. The genetic background reported in our previous study was used in the current study. MPSC1 cells, which were established from LGSOCs, were used in cell proliferation assays. We observed a higher ER expression in LGSOCs and SBTs than in SCAs (70%, 81%, and 50%, respectively). Thus, LGSOCs and SBTs exhibit higher ER expression than SCAs. Moreover, the *PIK3CA* mutation positively correlated with ER expression in LGSOCs (*p* = 0.0113). MPSC1 cells showed low ER expression on Western blotting. MPSC1 cell proliferation was significantly inhibited by fulvestrant (a selective ER downregulator). The activation of ER and PI3K/AKT signaling pathways may play an important role in LGSOC carcinogenesis. ER downregulation with fulvestrant or combination therapy with PI3K inhibitors is a possible novel treatment for patients with LGSOCs.

## 1. Introduction

Ovarian cancer is the third most lethal malignancy of the female reproductive tract, resulting in more than 14,070 deaths annually worldwide [1]. Over 95% of ovarian cancers are considered to be epithelial ovarian cancers, approximately 80% of which are serous carcinomas [2]. Currently, two main subtypes of serous ovarian carcinoma are identified: the common and well-characterized high-grade serous ovarian carcinomas (HGSOCs) with aggressive behavior, and the less common low-grade serous ovarian carcinomas (LGSOCs) with an indolent growth pattern [3]. Several studies have indicated numerous important differences between LGSOCs and HGSOCs in terms of molecular, histological, clinical, and epidemiological characteristics [4]. The classification of serous ovarian carcinomas into LGSOC and HGSOC is clinically significant, as patients with LGSOCs are known to have better progression-free survival than those with HGSOCs [5]. LGSOCs are thought to arise from serous cystadenomas or adenofibromas in a slow stepwise fashion; LGSOC development passes through serous borderline tumor (SBT) development. LGSOCs are typically diagnosed in younger patients and constitute less than 5% of all cases of ovarian carcinoma [6]. Despite the longer overall survival of patients with LGSOCs, LGSOCs are more resistant to standard cytotoxic chemotherapy than HGSOCs, with a response rate of 4% [7]. Therefore, understanding the alterations of molecular and signaling pathways that trigger oncogenesis in patients with LGSOCs is imperative for improving treatment outcomes by developing novel therapies. The complete mechanisms underlying LGSOC development are still not fully understood; however, the abundant expression of sex hormone receptors in LGSOCs suggests that estrogen receptor (ER) and progesterone receptor (PR) expression patterns may be associated with both tumor activity and prognosis [8,9,10]. A previous study showed that LGSOCs had a meaningful increase in ER (58% versus 27%, *p* = 0.003) and PR (43% versus 17%, *p* = 0.006) expression rates than HGSOCs [11]. However, only few studies have suggested the use of hormonal agents as possible treatment options for LGSOCs and ER+/PR+ breast cancer [12,13].

Additionally, higher frequencies of *KRAS*, *BRAF*, and *PIK3CA* mutations predict a dysregulation of the ERK and PI3K/AKT pathways, which are thought to be essential in the progression of LGSOCs [14,15,16]. Recently, we identified that approximately 60% of patients with LGSOC harbored *PIK3CA* mutations, which led to a continuous activation of PI3K/AKT pathways without *KRAS* mutation [16]. *PIK3CA* mutations are frequently observed in hormone receptor-positive breast cancers [17,18,19]. The abnormally activated PI3K/AKT pathway is less likely to respond to the use of chemotherapy agents, such as paclitaxel and trastuzumab, at both the preclinical and clinical stages of breast cancer similar to the LGSOCs [20,21]. Effective LGSOC control has been reported in some patients [22]; however, the clinical benefit of hormonal therapy in patients with *KRAS, BRAF*, *PIK3CA*, and *ERBB2* mutations remains unclear. Although hormonal therapy combined with PI3K/AKT pathway inhibitor use is an effective treatment for ER-positive breast cancer with *PIK3CA* mutation [23], the use of this synthetic lethal interaction therapy in women with progressive LGSOCs has not been reported. To fill this knowledge gap, we analyzed ER expression status in serous ovarian tumors, including LGSOCs, SBTs, and serous cystadenomas (SCAs); the relationship between ER status and oncogenic mutation status; and efficacy of ER inhibition, using MPSC1 cells obtained from LGSOCs. We found that the activation of both ER and PI3K/AKT signaling pathways may play an important role in LGSOC carcinogenesis; moreover, the downregulation of ER using fulvestrant alone or in combination with PI3K inhibitors is a possible novel treatment in patients with LGSOCs.

## 2. Materials and Methods

### 2.1. Study Population

We retrieved clinical samples from 10, 11, and 12 patients with LGSOC, SBT, and SCA, respectively, who were treated at Shimane University Hospital, Department of Obstetrics and Gynecology, Shimane Prefectural Central Hospital and Seirei Hamamatsu General Hospital from 2007 to 2017. All analyzed tissue samples were fixed with buffered formalin and embedded with paraffin blocks. The mean ages of patients with LGSOCs, SBTs, and SCAs were 48.1 (range, 26–83), 47.6 (range, 25–66), and 60.5 (25–81) years, respectively. An expert pathologist performed pathological diagnosis using hematoxylin and eosin-stained tissue sections. Histological tumor categorization was performed according to the World Health Organization tumor criteria, and tumor staging was based on the International Federation of Gynecology and Obstetrics classification system. All patients with LGSOCs and SBTs were primarily treated with cytoreductive surgery (including total abdominal hysterectomy, bilateral salpingo-oophorectomy, and omentectomy), with or without pelvic and para-aortic lymph node dissection, and adjuvant taxane-platinum combined chemotherapy. Patients with SCAs were treated with bilateral (or unilateral) salpingo-oophorectomy.

### 2.2. Immunohistochemistry

**Figure 1:**
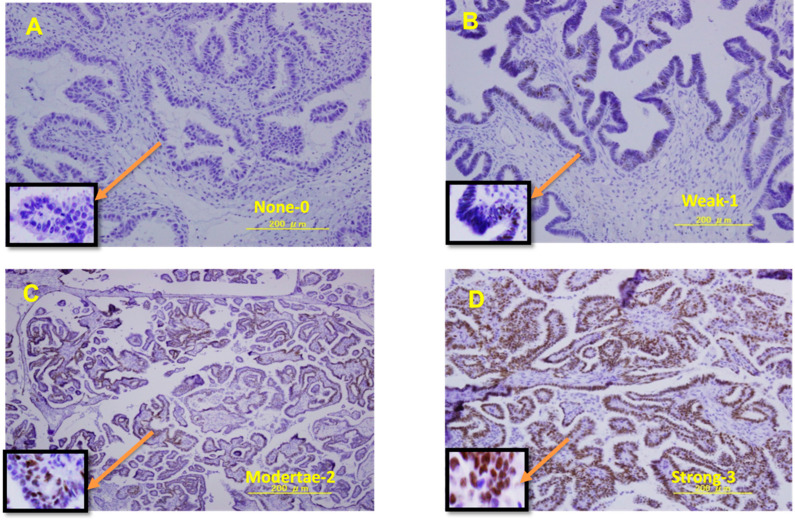
Representative image of ER immunostaining in LGSOCs. (**A**) Negative ER expression in LGSOCs; (**B**) weak ER expression in LGSOCs; (**C**) moderate ER expression in LGSOCs; (**D**) strong ER expression in LGSOCs. ER, estrogen receptor; LGSOCs, low-grade serous ovarian carcinomas.

Paraffin-embedded tissue sections were stained with ERα (D8H8) antibodies, purchased from Cell Signaling Technology (Danvers, MA, USA), following the manufacturer’s instructions. Briefly, paraffin slides were warmed, de-paraffinized with xylene, rehydrated in 100% to 70% graded ethanol, and finally washed with water. Subsequently, the slides were incubated for 20 min in phosphate-buffered saline (PBS) with 3% H_2_O_2_ and rinsed thrice with PBS. After antigen retrieval in a sodium citrate buffer, the slides were incubated overnight at 4 °C with ERα antibodies at a dilution of 1:200. Ovarian cancer tissue sections were used as negative controls, whereas the breast cancer cell line, T47D, was used as a positive control for ER expression. ER expression grading in epithelial cells was evaluated using the Histo score (H-score) system with the following equation: H-score $=\sum \mathrm{Pi}\left(i+1\right)$, where i is the epithelial cell intensity (0, negative; 1+, weak; 2+, moderate; or 3+, strong) and Pi is the percentage of stained tumor cells for each intensity, varying from 0% to 100%. For each case, 1000 cells were assessed in three or four different fields at a magnification of ×400. Two researchers who were blinded to the results (S.K. and K.N.) were involved in interpreting the stained slides. Representative photomicrographs of LGSOC tissues exhibiting positive and negative staining for the ER antigen are shown in Figure 1.

### 2.3. Cell Culture 

The human LGSOC cell line, MPSC1, was used in this study as an experimental cell line; the breast cancer cell line, T47D (ER-positive), was used as a positive control; and ovarian clear cell line, TOV-21G (ER-negative), was used as a negative control (Figure S1). The MPSC1 cell line was gifted by Dr. Ie-Ming Shih (Johns Hopkins Medical Institutions, Baltimore, MD, USA). T47D and TOV-21G cell lines were obtained from the American Tissue Culture Center (Rockville, MD, USA). All cell lines were seeded in T75 flasks at 1 × 10^6^ cells/mL in Dulbecco’s Modified Eagle Medium (Life Technologies, Gaithersburg, MD, USA), supplemented with 10% fetal bovine serum and 1% penicillin/streptomycin, and maintained at 37 °C in a humidified atmosphere with 95% air and 5% CO_2_. The culture medium was changed routinely every 2 days. In our experiments, we cultured an equal number of cells for each cell line; the number of cells was determined using a cell counting machine, and the cell doubling times of the MPSC1, T47D, and TOV-21G cell lines were 54 h 55 min, 62 h 49 min, and 21 h 19 min, respectively.

### 2.4. Western Blot Analysis

Sample cell lysis solutions were prepared using RIPA buffer (PBS, 1% NP-40, 0.5% sodium deoxycholate, and 0.1% sodium dodecyl-sulfate) containing 0.1 mg/mL phenylmethyl sulfonyl fluoride, 30 mg/mL aprotinin, and 1 mM sodium orthovanadate. The cells were scraped using a cell scraper under ice for 20 s. The cell lysis solution was transferred into Eppen tubes after pipetting the cell pellets 15 times. Moreover, the cell lysates were sonicated in an ice bath sonicator for 20 min, and then centrifuged at 14,000× *g* in a pre-cooled centrifuge for 15 min. Protein concentration in the cell lysates was measured using the Bradford method, and 10 µg of denatured protein was loaded per well for gel electrophoresis. Western blot analysis was performed using the sodium dodecyl sulphate-polyacrylamide gel electrophoresis system, as previously described [24]. Protein samples were loaded, electrophoresed using 10% tris-glycine-sodium dodecyl sulphate polyacrylamide gel (Invitrogen, Carlsbad, CA, USA), and transferred to polyvinylidene difluoride membranes via Bio-Rad semi-dry trans blotters (TransBlot^®^ SD cell, Hercules, CA, USA). The membranes were blocked with LI-COR blocking buffer (LI-COR, Lincoln, NE, USA) for 1 h at room temperature (25 °C). Thereafter, primary ERα antibodies were added (dilution 1:100, Abcam, San Jose, CA, USA), and the membranes were incubated overnight at 4 °C on a shaker. The next day, the primary antibody solution was discarded, and the membrane was washed with 0.1% PBS-T four times for 5 min each. The membrane was placed in secondary antibodies (goat anti-rabbit 1:10,000, horseradish peroxidase conjugated IgG) for 1 h at room temperature (25 °C) and subsequently washed with 0.1% PBS-T four times for 5 min each. Western blot images were visualized via enhanced chemiluminescence from a ChemiDoc™ TOUCH scanner.

### 2.5. Cell Proliferation Assay

Approximately 3000 cells per well in 180 μL of media were cultured in a 96-well plate. After 2 days of incubation, the growth medium was discarded and replaced with an experimental medium of various concentrations (10, 100, and 1000 nM) of fulvestrant for 96 h. Fulvestrant was purchased from Sigma-Aldrich (St. Louis, MO, USA). After treatment with the drug for the indicated durations, cells were treated with 3-(4,5-dimethylthiazol-2-yl)-2, 5-diphenyl tetrazolium bromide (MTT) solution (5 mg/mL in PBS) and further incubated for 4 h at 37 °C. After the supernatant was replaced with 100 μL of dimethyl sulfoxide, the absorbance was read at 490 nm using a 96-well plate reader (Bio-Rad, Winooski, VT, USA). “Percent of cell survival” was defined as the relative absorbance of treated versus untreated cells. Viability assays were performed in three independent experiments.

### 2.6. siRNA Transfection

The siRNA preparation and RNA interference were performed over 72 h using lipofectamine^®^ RNAiMAX (Invitrogen, Carslsbad, CA, USA), according to the manufacturer’s protocol. The effects of siRNA ERα (sc-2395 Santa Cruz Biotechnology, Santa Cruz, CA, USA) were compared with those of control siRNA-infected cells. Briefly, 50,000 cells were placed into 6-well plates and allowed to grow to a confluent monolayer for transfection. The day after confluence, transfection was performed by mixing Opti-MEN^®^ medium (150 μL) with Lipofectamine (9 μL) and Opti-MEN^®^ medium (150 μL) with ERα siRNA (6 μL (20 nM)). Subsequently, these two diluents were mixed in a ratio of 1:1 and kept at room temperature for 5 min; finally, 250 μL of mixed siRNA was added into each well. After 72 h of transfection, the cells were harvested for reverse transcription polymerase chain reaction (RT-PCR) to measure the effect of siRNA. Thereafter, MTT assay was performed to observe the cell proliferation ability with ERα knockdown.

### 2.7. Reverse Transcription Polymerase Chain Reaction

QIAGEN buffer RLT (QIAGEN GmbH, QIAGEN, Hidden, Germany) was used to homogenize the cultured cell pellets. Total RNA was extracted using a standard protocol according to the manufacturer’s instructions, and RNA quantification was performed using a spectrophotometer (NanoDrop ND-1000, NanoDrop Technologies, Wilmington, DE, USA). First-strand cDNA synthesis and amplification were performed using reverse transcription reagents (Thermo Fisher Scientific, Inc., Waltham, MA, USA), according to the manufacturer’s guidelines. The PCR involved 7.6 μL of cDNA and 12.4 μL of Syber Green Master Mix with pair primers. The primer designs used in RT-PCR were ERα forward primer 5′-TGG GCT TAC TGA CCA ACC TG-3′ and reverse primer 5′-CCT GAT CAT GGA GGG TCA AA-3′. The standard PCR conditions were set as follows: 95 °C for 30 s, 95 °C for 5 s, 60 °C for 30 s; initial denaturation followed by 40 cycles of amplification at 95 °C for 15 s, 60 °C for 30 s, and 95 °C for 15 s. The data were normalized to GAPDH levels and expressed as relative mRNA levels. The experiment was repeated independently at least three times.

### 2.8. Statistcal Analysis

Continuous variables in this study are expressed as mean ± standard deviation. Statistical analysis was performed using SPSS version 21.0 (SPSS Inc., Chicago, IL, USA). Student’s *t*-test (for comparison of two groups) and Pearson’s chi-square test were performed to compare differences between experimental groups. Statistical significance was defined as a two-sided *p* value *<* 0.05.

## 3. Results

### 3.1. ER Expression Was Higher in LGSOCs and SBTs Than in SCAs

**Figure 2:**
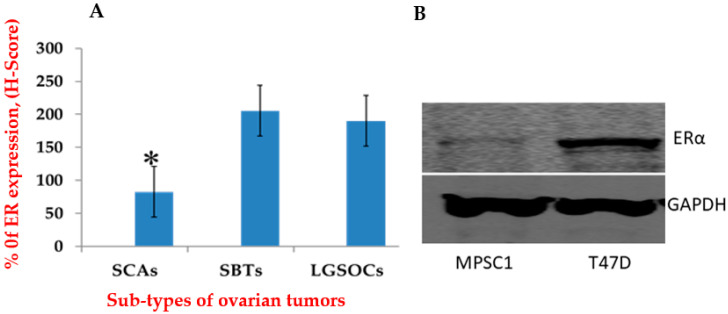
ER expression levels in ovarian serous tumors and MPSC1 cells. (**A**) ER expression levels in SCAs, SBTs, and LGSOCs. The expression level of ER in SBTs and LGSOCs is significantly (* *p* < 0.05) higher than that in SCAs; however, ER expression variation was not significant between SBTs and LGSOCs. (**B**) ER expression by Western blotting in MPSC1 and T47D cells. Uncropped Western Blot of ERα expression and a bar graph of Western Blot analysis we can be found at Figure S2. ER, estrogen receptor; SCAs, serous cystadenomas; SBTs, serous borderline tumors.

To clarify the prospective role of sex steroid hormones in the invasive progression of precancerous lesions to cancer, we evaluated the hormone receptor profile in LGSOCs, SBTs, and SCAs. Mean values of variables were used as cut-offs to dichotomize the data into positive and negative. The frequency of ER expression was 70% (7/10) in LGSOCs, 82% (9/11) in SBTs, and 50% (6/12) in SCAs. We compared the means of continuous variables for ER expression (Figure 2A); LGSOCs and SBTs showed significantly higher ER expressions than SCAs (*p* = 0.001). However, LGSOCs and SBTs did not show any significant variation in ER expression (*p* = 0.762).

### 3.2. ER Expressions in MPSC1

To examine the putative action of estrogens in LGSOCs, we first analyzed ER expression via Western blot analyses of MPSC1 and T47D cells (positive control). MPSC1 cells showed low ER expression whereas T47D cells showed high ER expression (Figure 2B). TOV-21G cells showed no ER expression (data not shown).

### 3.3. Regulation of Cell Growth Depends on ER Expression in MPSC1 Cells

**Figure 3:**
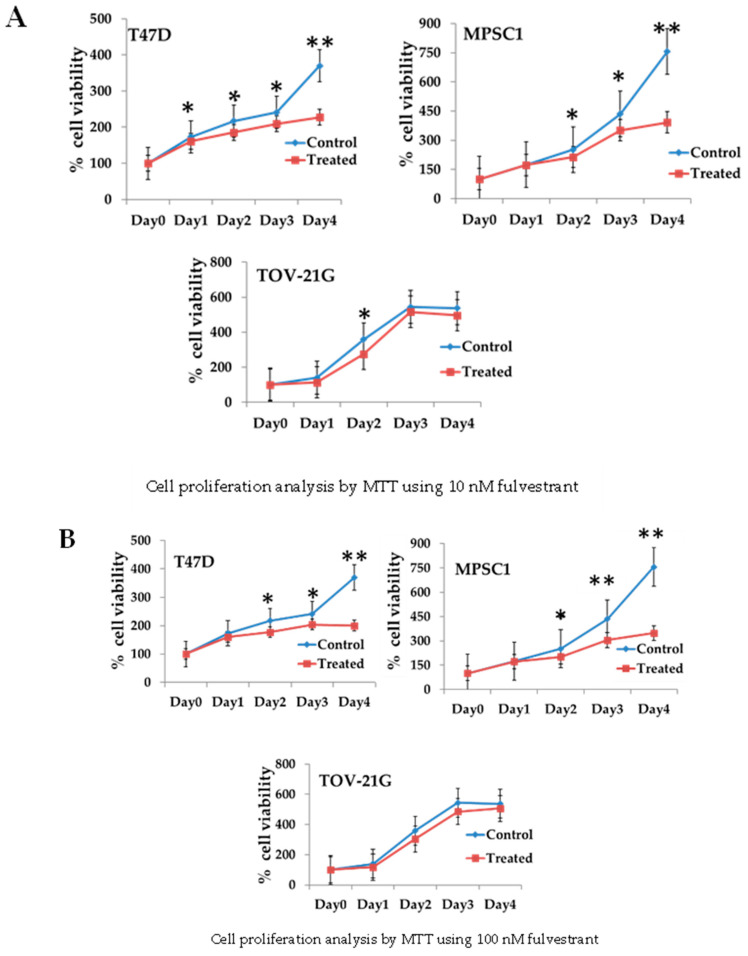

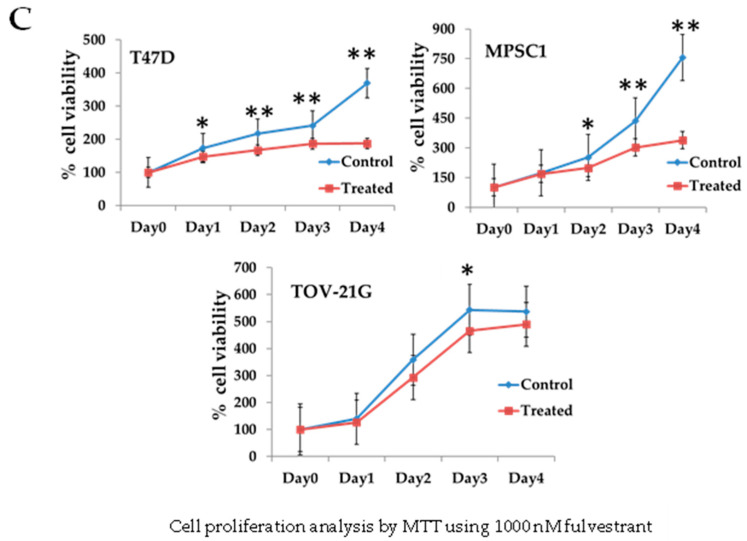
Cell proliferation analysis using fulvestrant in MPSC1, T47D, and TOV-21G cell lines. Different fulvestrant concentrations: (**A**) 10 nM; (**B**) 100 nM; and (**C**) 1000 nM. MTT assays in the T47D and MPSC1 cell lines showed significant (* *p* < 0.05; ** *p* < 0.01) growth inhibiting ability compared to that in the control cell line. The experiment was conducted in triplicate, and data are presented as mean ± standard deviation. MTT, 3-(4,5-dimethylthiazol-2-yl)-2,5-diphenyl tetrazolium bromide.

To examine the necessity of activated ERs for the proliferation of LGSOC cells, MPSC1 cell proliferation was evaluated 96 h following treatment with fulvestrant, a selective ER antagonist. Cell growth was significantly reduced in MPSC1 cells treated with fulvestrant at 10, 100, and 1000 nM than in untreated MPSC1 cells (Figure 3). This result suggests that ER has a great effect on LGSOC cell proliferation. T47D and TOV-21G cells, which were used as positive and negative controls, respectively, showed results that were consistent with those in the literature (Figure 3) [25,26]. Overall, our findings indicate that fulvestrant use could be a therapeutic option in patients with LGSOCs.

### 3.4. Relationship between Different Oncogenic Mutations and Hormone Receptors

We previously created oncogenic mutation profiles in LGSOCs [16]. In the present study, *PIK3CA* showed the highest mutation frequency (60%), followed by *ERBB2* (30%), *BRAF* (20%), and *KRAS* (0%) (Table 1) [16]. Interestingly, there was a significant association between positive ER expression and *PIK3CA* mutation (*p* = 0.011), whereas *BRAF* and *ERBB* mutations did not reveal any significant relationship with ER expression (*p* = 0.300 and *p* = 0.097, respectively) (Table 2). There were no significant associations among ER expression status, age, and International Federation of Gynecology and Obstetrics stage (Table 2).

### 3.5. siRNA Knockdown of ERα Decreased ERα Expression in MPSC1 Cells as Shown by RT-PCR

To confirm the effects of the ER on MPSC1 cells, the ERα underwent ERα siRNA knockdown. RT-PCR analysis revealed that ERα siRNA knockdown significantly suppressed mRNA ERα expression in the MPSC1 cell line (*p* < 0.001) (Figure S3). The T47D cell line was used as a positive control (*p* < 0.002). GAPDH expression was used as an internal control for the cDNA input.

### 3.6. ERα Knockdown Decreases Cell Proliferation in the MPSC1 Cell Line

To confirm the effects of ER expression on MPSC1 cell proliferation, we applied a complementary approach using a gene knockdown system to reduce ER expression in the MPSC1 cell line. Figure S4 shows that ERα siRNA knockdown significantly inhibited MPSC1 cell proliferation ability after 72 h in contrast to the control siRNA group (79.05 ± 0.0175% vs. 179.89 ± 0.025%, *p* < 0.001).

## 4. Discussion

LGSOC is an atypical histologic subtype of ovarian carcinoma with distinct clinical features; moreover, the rate of resistance to conventional chemotherapy is high, with an extended overall survival of patients with LGSOCs. LGSOC accounts for more than 10% of serous carcinomas [6]. Women typically present LGSOC at a younger age and have an indolent clinical course compared with that of high-grade serous ovarian cancers. A previous study reported that ovarian malignancies in children and adolescents accounted for 10–20% of all ovarian masses [27]. Most ovarian cysts in children are benign and self-resolving. However, if malignancy is identified, it is usually necessary to perform surgery with a wide range of interventions [28]. The primary treatment of LGSOC is the same as that of other epithelial ovarian cancer subtypes and comprises debulking surgery and platinum/taxane-based chemotherapy; however, the use of platinum-based chemotherapy is debated due to low response rates in patients with LGSOCs. Several recommendations can be followed to preserve the fertility of young patients with LGSOCs: (1) removing the whole body of the tumor, (2) sparing the fallopian tube without filmy and dense adhesions, (3) collecting ascitic fluid for cytology, (4) removing areas with suspected tumor invasion, and (5) examining the iliac and aortocaval nodes and biopsying areas with suspected tumor invasion [28]. In cases of tumor recurrence, the use of hormonal therapies, bevacizumab, and targeted therapies such as MEK inhibitors may offer benefits in combination with cytotoxic chemotherapy.

Several studies have described ER and PR expressions in breast, endometrial, and prostate cancers [29,30]. In the current study, we assessed ER expression in LGSOCs, SBTs, and SCAs using immunohistochemistry. LGSOCs are often associated with SBTs, according to the World Health Organization classification, due to their non-aggressive behavior. However, few SBTs (<10%) reportedly evolve to LGSOCs [31,32] with uncommon micropapillary subtypes; thus, patients with SBTs are predisposed to developing LGSOCs [31]. Constitutive ER expression was observed in SBTs in the current study as well as in a previous report [33]. It remains unclear whether carcinogens for LGSOCs from SBTs or SCAs are affected by hormone status; hence, we considered focusing on the examination of steroid hormone receptor profiles in serous ovarian tumors, including LGSOCs, SBTs, and SCAs. In the current study, LGSOC and SBT cells were ER-positive in 70% and 81% of cases, respectively. Taken together, present and previous study findings suggest that the higher ER expressions in LGSOCs and SBTs compared to that in SCAs are responsible for malignant transformation [34]. Mechanistic studies have supported the proliferative and negative apoptotic role of the ER [35], and thus we speculated that the high ER expression in LGSOCs may constitute an evolving mechanism for tumor proliferation. Recent studies concluded that conventional (neoadjuvant and adjuvant) chemotherapy is of little or no benefit in LGSOC treatment compared with HGSOC treatment [36,37,38]. These studies reported that at the completion of primary chemotherapy (platinum-based) in women with stage II to IV LGSOCs [36,38,39], more than 40% of women showed tumor persistence, and response rates were less than 5% in both neoadjuvant and salvage chemotherapy settings [40]. A recent study, involving approximately 5114 patients who randomly participated four times in phase III trials conducted by the *German Gynecological Oncology Group* and metadata base, demonstrated that the chemotherapy response rate in patients with sub-optimally debunked LGSOCs was significantly lower than that in patients with sub-optimally debunked HGSOCs (23% vs. 90%, respectively) [41]. Despite the low response rate to platinum/taxane-based chemotherapy in patients with LGSOCs, this combination remains the standard regimen in clinical situations. Although LGSOCs are usually chemoresistant, they are not entirely resistant to chemotherapy, and good responses have been observed in some women with LGSOCs [37,40,42]. In addition, many women with LGSOCs maintain stable disease conditions for a long period of time [37,40]. It is yet to be determined whether this stable disease condition is a consequence of the indolent behavior of LGSOCs or the advantageous effect of chemotherapy. However, the fact that LGSOCs are relatively chemoresistant has stimulated the search for alternative therapies. Hitherto, different treatment options such as bevacizumab, PARP inhibitors [43], or hyperthermic intraperitoneal chemotherapy [44] have been used to increase survival in patients with ovarian cancer (including LGSOCs). However, the results obtained are not completely satisfactory. Hormonal therapy may play an important role in the successful treatment of women with LGSOCs [8,45]. A previous study reported ER+/PR+, ER+/PR, ER−/PR+, and ER-/PR- expressions of 21.8%, 17.4%, 13.0%, and 47.8%, respectively, among 27 women with LGSOCs; this study presumed that only a subgroup of tumors, which expressed dual steroid receptor might respond to hormonal treatment [8]. Patients with breast cancer who received bortizomib and fulvestrant, a hormonal agent, showed significantly prolonged progression-free survival [46]. Based on the results of the clinical trial conducted by the MD Anderson Cancer Center group, the use of hormone therapies was considered as part of the management of relapsed LGSOCs, with overall hormonal therapy response and disease control rates of 9% and 62%, respectively [47]. A recent meta-analysis of studies on estrogen-based endocrine replacement therapy established a statistically significant increase in the occurrence of epithelial ovarian cancer among women receiving hormone replacement treatment [48]. A previous study reported that an administration of a small dose of any estrogen type could induce different ovarian changes such as the development of cystadenomas, neoplastic tumors, and papillary excrescences in a mammalian model [49].

The use of fulvestrant, a pure Food and Drug Administration-approved ER antagonist devoid of agonist activities, is advantageous for hormone sensitive patients with advanced breast cancer, who have received high-dose chemotherapy [50]. The 17-β estradiol derivative is replaced with an alkyl chain at the 7-α position, which confers on fulvestrant a 100-fold greater binding strength than tamoxifen and more efficacy in the inhibition of estrogen signaling than either tamoxifen or aromatase inhibitors [51,52]. A randomized multicenter phase III clinical trial was conducted to evaluate and compare the efficacies of fulvestrant and exemestane in 693 patients with advanced breast cancer experiencing postmenopausal changes. This was done to compare the potency and acceptability of fulvestrant with that of the firmly established third-generation aromatase inhibitor (anastrozole). However, fulvestrant did not show significant dominance over anastrozole, as the duration of response in the study revealed that fulvestrant was as potent as anastrozole [52]. In vitro analysis showed that the use of fulvestrant alone disrupted the proliferation of MCF-7 cells [53]. An in vivo analysis showed that the use of S-1/fulvestrant combination therapy significantly increased antitumor activity compared to that of other hormonal monotherapies [54]. Furthermore, fulvestrant use reportedly reduces ER signaling by downregulating ERα levels. The combined effects of S-1 and fulvestrant on tumorigenic ERα-expressed MCF-7 cell lines are comparable to those of other combined endocrine therapies with an anti-estrogen agent (4-hydroxytamoxifen) and an aromatase inhibitor (anastrozole) [54]. These previous results are consistent with our study findings, wherein cell proliferation was significantly inhibited in fulvestrant-treated MPSC1 cells.

Previous studies from Western countries have reported that women with LGSOCs often harbor active gene mutations for *KRAS*, *BRAF*, and *ERBB2*, which are involved in the ERK pathway; however, our study population (a group of Japanese patients) presented with driver mutations for *PIK3CA*, which is linked to the AKT/mTOR pathway, as was previously reported [16]. In addition, our present study demonstrated a significant correlation between *PIK3CA* mutations and ER expression. Approximately 25% of breast cancer patients with *PIK3CA* mutations, which is one of the most common genetic aberrations in breast cancers, are ER-positive [55]. Moreover, there is sufficient evidence suggesting that the ER and mTOR signaling pathways interact at several levels, and involve overlapping signaling cascades and outputs [56,57]. Previous and current study findings support the hypothesis that interaction between mTOR and ER signaling may be required for LGSOC proliferation.

Fulvestrant has been used as a first-line treatment to meet the targets of endocrine therapy, including extending survival rates, maintaining the quality of life, and delaying the initiation of chemotherapy in patients with advanced ER-positive breast cancer [58]. Despite the established efficiency of hormonal therapy, one in three patients developed resistance in one study [59]. Another report revealed that an inhibition or a mitigation of estrogen synthesis via an obstruction of either the sulfatase or aromatase pathway has a therapeutic consequence on breast cancer [60]. Epithelial ovarian cancers frequently reveal ER expression and may respond to anti-estrogen therapy. A clinical trial in patients with relapsed ovarian cancer who received a single dose of fulvestrant showed an adequate tolerance to and efficacy of the drug [61]. In the present study, we observed that fulvestrant-treated MPSC1 cells had a lower proliferation activity than non-fulvestrant-treated MPSC1 cells, which corroborated with previous study findings [60,61]. Therefore, fulvestrant use may be an appropriate therapeutic option for patients with ER-positive LGSOCs.

This study has a major strength: it is the first molecular study demonstrating that the use of fulvestrant, a selective ER down regulator, is effective in LGSOC treatment. However, this study has a limitation. We did not observe the effect of fulvestrant on the ER expression grading of the clinical samples. Therefore, the use of three-dimensional cell cultures such as patient-derived cancer organoids, which closely mimic the in vivo conditions, is essential to confirming the current findings. Although we observed a significant association between positive ER expression and *PIK3CA* mutation, further research is required to analyze the sensitivity of PI3K inhibitors in ER-positive MPSC1 LGSOC cells in order to clarify the therapeutic potential of PI3K inhibitors.

## 5. Conclusions

Current in vitro evidence and immunohistochemical analysis of ER in LGSOCs showed that estrogen may regulate the development of LGSOCs. In addition, the ER is highly expressed in subsets of ovarian tumors with *PIK3CA* mutations. This study demonstrated that fulvestrant use significantly inhibited the growth of ER-positive MPSC1 LGSOC cells. Thus, we speculate that the use of fulvestrant, alone or in combination with PI3K inhibitors, may have clinical benefits; therefore, fulvestrant use is a therapeutic option for patients with ER-positive LGSOCs.

## Figures and Tables

**Table 1:** Expression status of ER and oncogenic mutation in LGSOCs.

No	Age	FIGO Stage	ER Expression	PIK3CA E9	PIK3CA E20	ERBB2	BRAF
1	37	Ⅲc	**+**	G1633C (E545Q)	WT	WT	WT
2	61	Ⅰc	**+**	WT	WT	A2384G (Q795R)	WT
3	61	IVb	**+**	G1633A (E545K)	WT	WT	WT
4	83	Ⅰa	**+**	A1634C (E545A)	WT	WT	WT
5	27	Ⅰc	**+**	A1634C (E545A)	WT	A2384G (Q795R)	T1796A (V600E)
6	61	Ⅲc	**-**	WT	WT	WT	WT
7	40	Ⅰc	**+**	A1634C (E545A)	WT	WT	T1796A (V600E)
8	48	Ⅰc	**-**	WT	WT	WT	WT
9	26	Ⅲc	**+**	G1633C (E545Q)	WT	WT	WT
10	37	IIc	-	WT	WT	A2384G (Q795R)	WT

ER, estrogen receptor; FIGO, International Federation of Gynecology and Obstetrics; LGSOC, low-grade serous ovarian carcinoma; **+**, Positive expression; **-,** Negative expression; WT, wild type.

**Table 2:** Association between ER expression and mutation status in patients with LGSOCs.

Factors	ER-Positive	ER-Negative	*p* Value
	n = 7	n = 3	
PIK3CA			
WT	1	3	0.0113
MT	6	0	
BRAF			
WT	5	3	0.300
MT	2	0	
ERBB			
WT	6	1	0.097
MT	1	2	
Age			
<60	5	1	0.259
>60	2	2	
FIGO stage			
I	4	1	0.491
II, III, IV	3	2	

MT, mutation.

## Data Availability

The data of the present study are available on request from the corresponding author (K.N.).

## Supplementary Materials

The following supporting information can be downloaded at: https://www.mdpi.com/article/10.3390/curroncol29060321/s1, Figure S1: Morphological characteristics of experimental cell lines (A) T47D (Breast cancer cell line), (B) MPSC1 (Low-grade serous ovarian cancer cell line), and (C) TOV-21G (Ovarian clear cell line); Figure S2: (A) Western blot analysis of ERα expression in MPSC1 and T47D cell lines with reference to the GAPDH protein. (B) Relative protein expressions of (I) ERα and (II) GAPDH in MPSC1 and T47D cell lines; Figure S3: mRNA expression level of ERα was evaluated in (A) MPSC1 and (B) T47D cell lines. RT-PCR results revealed that both cell lines (A,B) showed significantly lower ERα expression levels compared to siRNA control (*p* < 0.001 and *p* < 0.002, respectively); Figure S4: (A) T47D and (B) MPSC1 cell lines significantly reduce cellular proliferation at 20 nM dose of ERα siRNA, but (C) the TOV-21G cell line has no effect on cell growth compared to the si-control cell line.
