# Supplementary figures and images for: Promising Therapeutic Impact of a Selective Estrogen Receptor Downregulator, Fulvestrant, as Demonstrated In Vitro upon Low-Grade Serous Ovarian Carcinoma Cell Lines
Source: Curr Oncol. 2022 Jun 1;29(6):4020–33. doi: 10.3390/curroncol29060321 (PMC9221871; doi:10.3390/curroncol29060321)

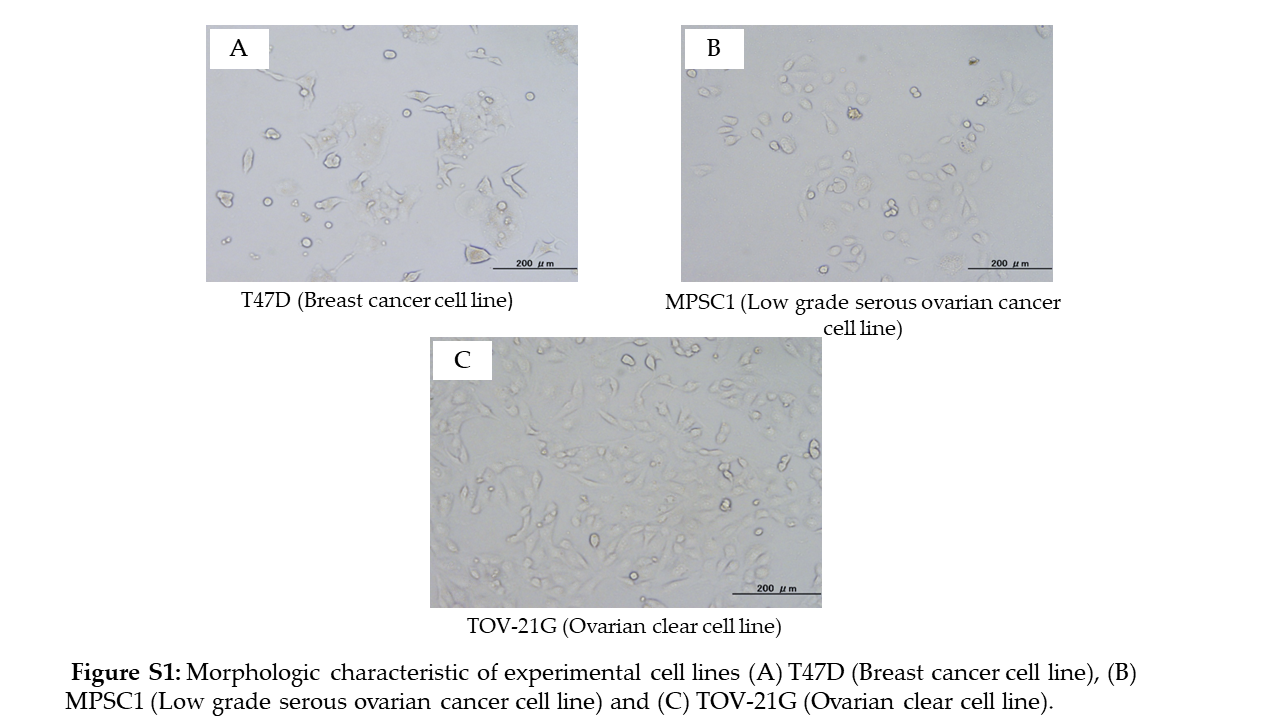

Supplement: Supplementary file 1 [file curroncol-29-00321-s001.zip › supplimentary figure for submission/Figure S1.TIF]

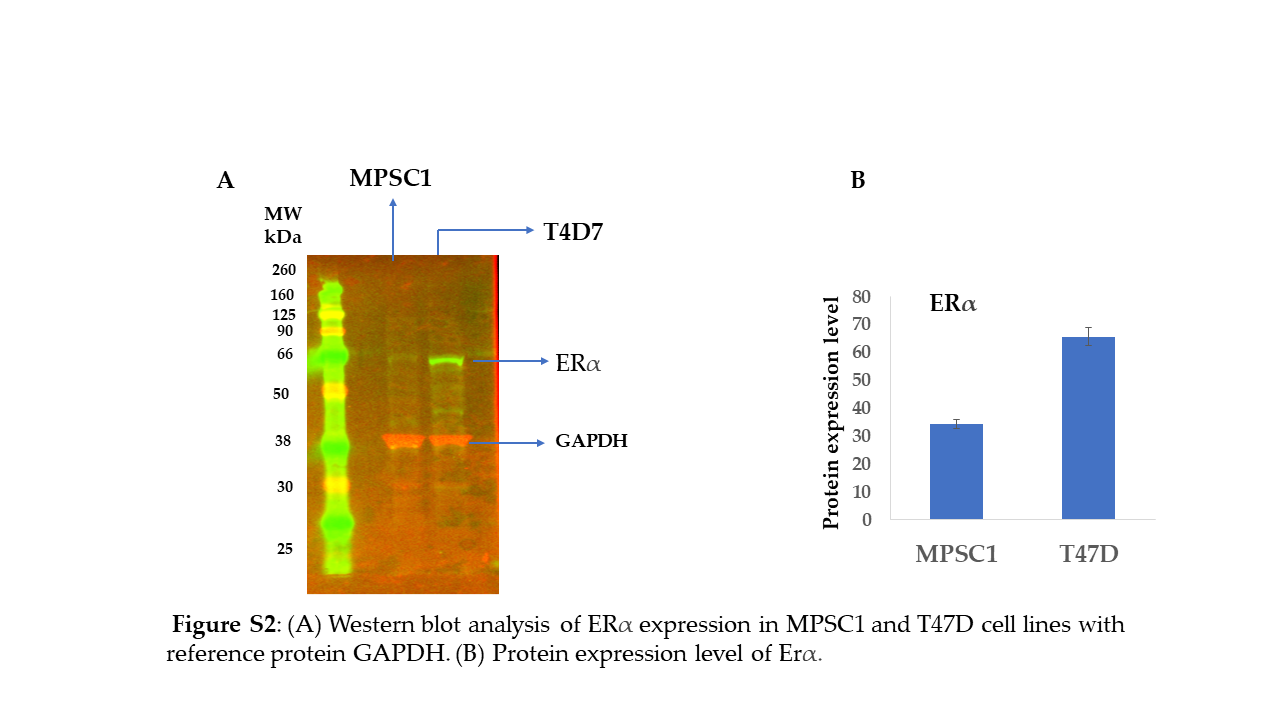

Supplement: Supplementary file 1 [file curroncol-29-00321-s001.zip › supplimentary figure for submission/Figure S2.TIF]

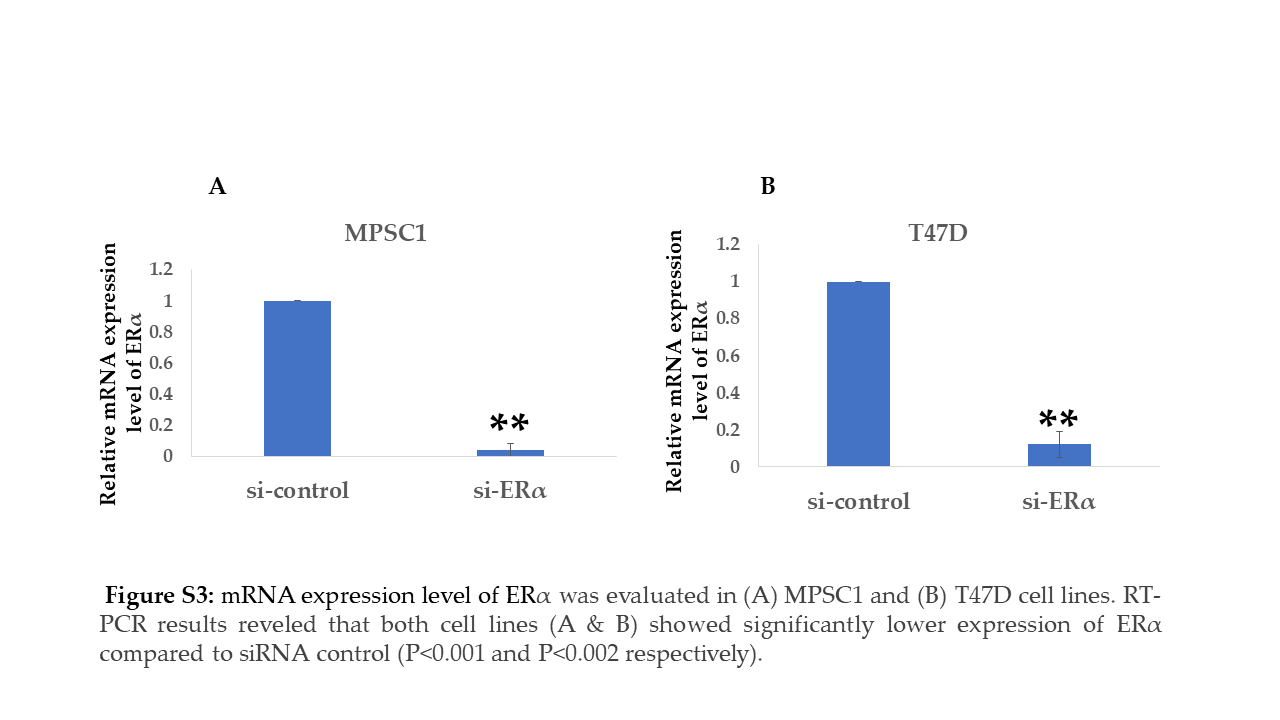

Supplement: Supplementary file 1 [file curroncol-29-00321-s001.zip › supplimentary figure for submission/Figure S3.TIF]

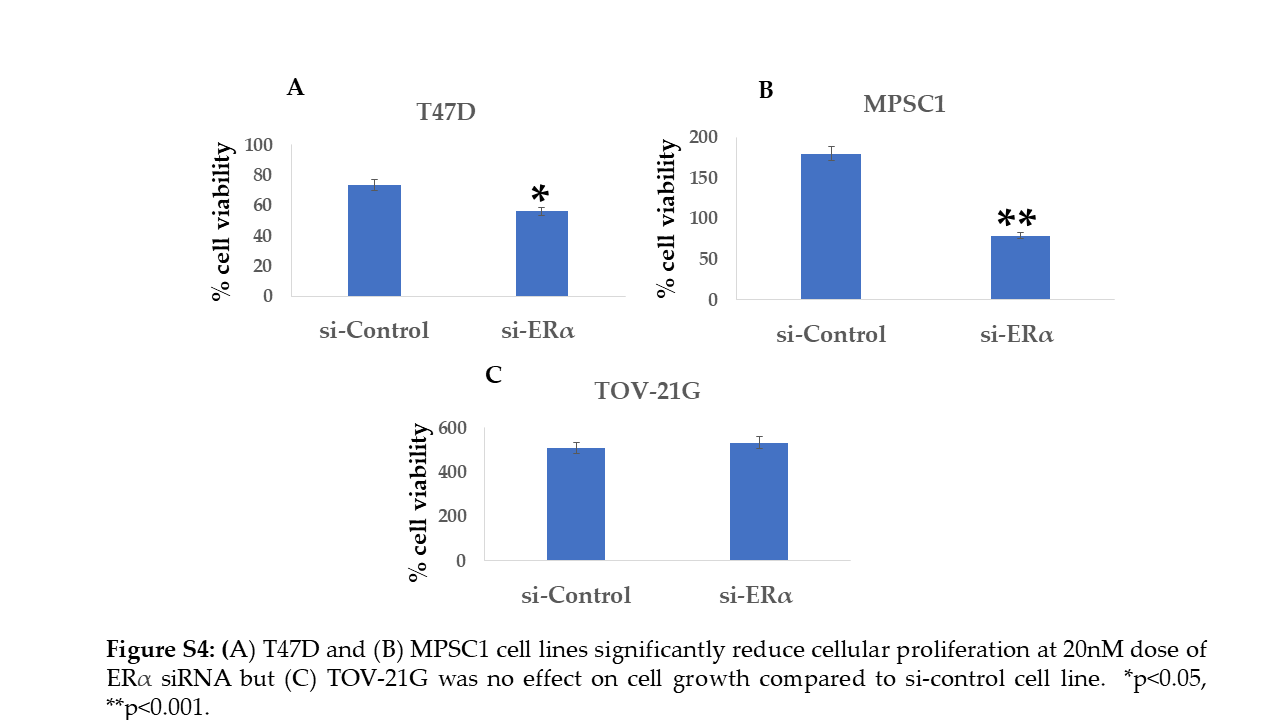

Supplement: Supplementary file 1 [file curroncol-29-00321-s001.zip › supplimentary figure for submission/Figure S4.TIF]
